# Ectodomain Pulling Combines with Fusion Peptide Inserting to Provide Cooperative Fusion for Influenza Virus and HIV

**DOI:** 10.3390/ijms21155411

**Published:** 2020-07-29

**Authors:** Sergey A. Akimov, Oleg V. Kondrashov, Joshua Zimmerberg, Oleg V. Batishchev

**Affiliations:** 1 Laboratory of Bioelectrochemistry, A.N. Frumkin Institute of Physical Chemistry and Electrochemistry, Russian Academy of Sciences, 31/4 Leninskiy Prospekt, 119071 Moscow, Russia; academicoleg@yandex.ru (O.V.K.); olegbati@gmail.com (O.V.B.); 2 Section on Integrative Biophysics, Eunice Kennedy Shriver National Institute of Child Health and Human Development, National Institutes of Health, Bethesda, MD 20892, USA; zimmerbj@mail.nih.gov

**Keywords:** membrane fusion, enveloped virus, influenza virus, human immunodeficiency virus (HIV), theory of elasticity, fusion rosette, cooperativity, fusion protein, membrane-mediated interaction

## Abstract

Enveloped viruses include the most dangerous human and animal pathogens, in particular coronavirus, influenza virus, and human immunodeficiency virus (HIV). For these viruses, receptor binding and entry are accomplished by a single viral envelope protein (termed the fusion protein), the structural changes of which trigger the remodeling and merger of the viral and target cellular membranes. The number of fusion proteins required for fusion activity is still under debate, and several studies report this value to range from 1 to 9 for type I fusion proteins. Here, we consider the earliest stage of viral fusion based on the continuum theory of membrane elasticity. We demonstrate that membrane deformations induced by the oblique insertion of amphipathic fusion peptides mediate the lateral interaction of these peptides and drive them to form into a symmetric fusion rosette. The pulling force produced by the structural rearrangements of the fusion protein ectodomains gives additional torque, which deforms the membrane and additionally stabilizes the symmetric fusion rosette, thus allowing a reduction in the number of fusion peptides needed for fusion. These findings can resolve the large range of published cooperativity indices for HIV, influenza, and other type I fusion proteins.

## 1. Introduction

Enveloped viruses, as a class, include top human killers such as influenza viruses, human immunodeficiency virus (HIV), coronaviruses, Ebola, and hepatitis C viruses [1]. Despite the differences in infection pathways and clinical manifestations, these viruses have similarities in their structure and lifecycle. The genetic material of enveloped viruses is wrapped into two shells. The first one is a protein capsid, or scaffold, which is covered by a lipid membrane layer. The lipid envelope of the virus includes surface glycoproteins [2]. In order to infect the cell, the virus should deliver its genetic material inside it by breaking two barriers: its own envelope and the plasma membrane of the target cell. That is why membrane fusion is a necessary step in the lifecycle of all enveloped viruses. As a result of membrane fusion, the viral interior and the cell cytoplasm unite, forming a continuous pathway for viral genetic material [3]. Unlike the fusion of cells, organelles, vesicles, etc., which usually requires large protein complexes and the consumption of energy [4,5], viral fusion is generally orchestrated by fusion proteins without ATP or GTP hydrolysis [3]. These proteins usually represent the oligomeric structures of several monomers, undergoing conformational rearrangements to bring viral and cellular membranes into close contact [3].

Fusion proteins are divided into three classes, among which proteins of the class I (e.g., influenza A virus hemagglutinin (HA) and HIV Env gp120/gp41 protein) are the best studied [2]. Despite the differences in the fold of fusion proteins from different classes, all of them have a transmembrane part (embedded into the viral lipid envelope), an ectodomain, and cytoplasmic tails with which they usually interact with the viral protein scaffold [6]. At the beginning of the infection cycle, fusion proteins bind specific receptor molecules on the cell surface. After some triggering event, which could be receptor binding (e.g., for HIV) or a pH change inside the endosome (e.g., for influenza virus), the fusion protein attacks the cellular membrane with the fusion peptide, which is a specific N-terminal sequence of about 20 amino acids [6]. The fusion peptide goes out from the hydrophobic pocket of the fusion protein ectodomain to incorporate into the lipid bilayer of the target membrane as an effective anchor. In the ensuing course of continuing conformational rearrangements, the fusion protein refolds back, pulling the target cellular membrane with the incorporated fusion peptide toward the viral membrane [7]. Upon coming into close contact, these membranes merge, resulting in formation of the fusion pore, via which the viral genome can release into the cytoplasm. At this stage, the fusion protein folds into a post-fusion state, where its transmembrane part contacts with the fusion peptide [3]. If the fusion peptide does not reach the target membrane, it can incorporate into the viral membrane close to the transmembrane domain of the fusion protein, leading the fusion protein to the same post-fusion state but with its fusion peptide and transmembrane domain residing in the same (viral) membrane close to each other. The height of the post-fusion folded fusion protein is about 10 nm [8].

The fusion of viral and cellular membranes, driven by fusion proteins, goes through several sequential stages [9]. Fusion peptides are mostly amphipathic [10,11]. They are able to partially incorporate into the target membrane, simultaneously providing an anchor and lever for the application of forces and torques and locally deforming the target membrane. The depth of insertion of the fusion peptide regulates its fusogenic activity [11]. However, substitution of the transmembrane part of the fusion protein to the lipidic anchor hinders enlargement of the fusion pore [12,13]. Therefore, both the fusion peptide and transmembrane domain are necessary for effective fusion. Fusion proteins enforce the formation of opposing bulges on two membranes. Strong hydration repulsion acting on the tops of these bulges leads to the lateral displacement of polar lipid heads from the area of tight contact, resulting in the formation of hydrophobic patches, where such repulsion becomes locally reduced [14,15,16]. With some probability, these local convexes of opposing membranes merge, forming hourglass-shaped structures called stalks [16]. Experiments with fluorescently labeled lipids and water-soluble dyes show that the lipid flux precedes the mixing of the cytoplasm with viral interior [17]. This means that the merger of membranes should occur before the formation of the fusion pore. This stage is called hemifusion [18]. For influenza A virus, fusion may be arrested at the hemifusion state by a decrease of the temperature down to 4 °C [18]. The rate of the process is also lipid-dependent [18]. In particular, the positive spontaneous curvature of the contact monolayers of fusing membranes (achieved by, e.g., the addition of lysolipids) is shown to decelerate transition to the hemifusion, while negative spontaneous curvature (induced by addition of, e.g., cholesterol or dioleoylposphatidylethanolamine, DOPE) promotes it [19]. The positive spontaneous curvature in distal monolayers accelerates the transition of hemifusion to complete fusion, i.e., to the fusion pore formation, but it does not influence the rate of transition to the hemifusion state [18]. Basing on these data, in numerous works, it is proposed that the role of fusion proteins is merely to bring the membranes into close contact, while the rate of the rest of the fusion process is mainly determined by the energy of elastic deformations of fusing lipid bilayers [20]. Multiple theoretical models are devoted to estimation of the elastic energy of intermediate states of the fusion process [20,21,22,23]. It is shown that the main energy barrier of hemifusion strongly depends on the distance between two fusing membranes: the larger the distance, the higher the barrier [20,21]. The equilibrium intermembrane distance far from the fusion site is determined by the thickness of the layer of folded fusion proteins, whose fusion peptides do not reach the target cellular membrane. For hemagglutinin, this thickness is about 10 nm, as observed by electron microscopy [8]. Thus, the fusion proteins should locally overcome this distance to bring merging membranes together over a small area of contact, ensuring a relatively low height of the energy barrier for hemifusion. According to different estimations, the energy required for such a rearrangement of membranes is of the order of tens of *k_B_T* (where *k_B_* is the Boltzmann constant, *T* approximately 300 K, and thus *k_B_T* approximately 4.14 × 10^−19^ J) [20,21,22]. For comparison, the energy stored in a single trimer of hemagglutinin is about 60 *k_B_T*, i.e., 20 *k_B_T* per hemagglutinin monomer [24]. The same energy estimate is valid for HIV gp41 trimer [25]. This means that the effective membrane merger should require the cooperative action of several fusion proteins. For influenza-mediated fusion, the necessary number of hemagglutinin trimers in a fusion rosette is estimated as 3–9 [26,27]. However, for the case of gp41 of HIV, it is shown that a single trimer might be enough to complete fusion [27]. Thus, the energy stored in a single trimer of the fusion peptide does not completely determine the structure of the fusion rosette.

In fact, exceeding the critical surface density of fusion proteins on the viral membrane does not imply any cooperativity of their action, since the physical mechanisms ensuring the orchestrated release of mechanical energy stored in fusion proteins comprising the rosette are not clear. Without outlining such mechanisms, the forces and torques generated by different viral fusion proteins (hemagglutinins of influenza virus or gp120/gp41 proteins of HIV) cannot be considered additive, as they could be applied in random directions and might even partially compensate for each other, thus hindering fusion instead of promoting it. The mechanisms of the fusion rosette gathering are not clearly known at the moment. In principle, it could be driven by direct chemical protein–protein interactions. However, to the best of our knowledge, the probable sites of such interactions are not identified.

A specific functional feature of type I fusion proteins is a strong deformation of the target membrane; the viral membrane is deformed to a lesser extent because of the rigid envelope of the matrix protein underlying the lipid membrane [8]. The deformation of the target membrane is provided by incorporated fusion peptides. Elastic membrane deformations may laterally span over several to tens of nanometers [28,29,30]. Deformations induced by far-separated fusion peptides are independent, and their energies are additive. Upon approach of the peptides, these deformations overlap, resulting in effective lateral interaction. Deformation-mediated interactions were considered in numerous theoretical works [31,32,33]. The dependence of the energy of two membrane-incorporated peptides or proteins on the distance between them may correspond to repulsive, attractive, or complex non-monotonous interactions [34,35,36]. However, the distance between viral fusion proteins is not a variable, as their inner-virion domains are bound to the protein envelope via cytoplasmic tails, and thus the lateral positions of transmembrane domains are rather fixed [37,38,39]. Besides, the folding of fusion proteins during the conformational transition should result in a laterally anisotropic deformation of the target membrane. This means that when investigating the formation of a cooperative fusion rosette, one should consider the dependence of the elastic energy on the mutual orientation of fusion peptides, rather than on the distance between them. Under conditions of laterally immobile peptides, their mutual orientation is the parameter that determines the cooperative action of fusion proteins.

The fusion peptides of influenza hemagglutinin and HIV gp41 protein are insoluble in water [11,40,41]. In experiments studying the interactions of fusion peptides with model membranes, modified peptides are usually used, which are appended by a highly charged group [42] or amino acid sequence [43] connected to the C-terminal of the peptide either directly or via some flexible linker. For influenza, it is shown that such modified peptides shallowly incorporate into the model membrane, attaining a boomerang-like configuration with a small tilt angle with respect to the membrane plane [42]. In the case of HIV, fusion peptides insert deeper into the target membrane, still being amphipathic [11]. During the conformational transition, the ectodomains of type I fusion proteins most probably exert a pulling force and a torque on the fusion peptide anchored in the target membrane. This should lead to an increase of the tilt-angle of the peptide axis with respect to the membrane plane; i.e., the peptide should attain a somewhat oblique orientation and induce strongly laterally anisotropic deformations of the target membrane.

In the present work, we considered the interactions of laterally immobile obliquely oriented fusion peptides of type I fusion proteins exemplified by HIV and influenza A virus fusion proteins, which are mediated by the elastic deformations of the membrane. The peptides were incorporated into only one lipid monolayer of the target membrane. Instances of two and three interacting peptides were analyzed. By explicitly calculating the elastic energy of different configurations of the peptides, we demonstrated that membrane deformations drive three oblique peptides into a configuration possessing a central symmetry, which is necessary to form a cooperative fusion rosette. However, for two peptides, this symmetric configuration is achieved only when they are subjected to a pulling force, which is directed perpendicularly to the plane of the target membrane. The results obtained imply that elastic deformations of the target membrane induced by incorporated fusion peptides are capable of gathering the fusion proteins into a cooperative symmetric fusion rosette.

## 2. Results

### 2.1. Setting up the System

#### 2.1.1. Approximation of Fusion Peptide Trimer by Single Cylindrical Membrane Inclusion

**Figure 1:**
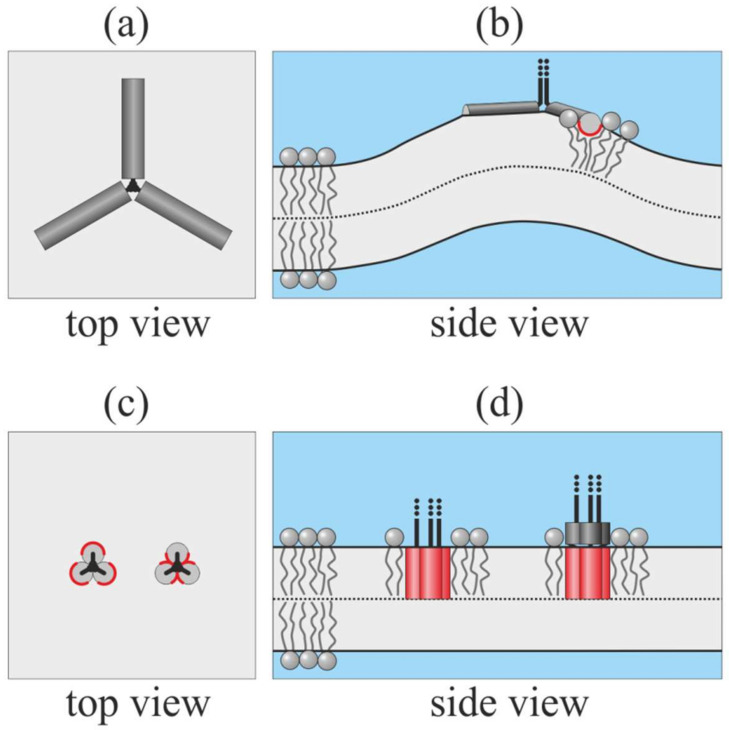
Possible ways of insertion of fusion peptides from one trimer of the type I fusion protein into the target membrane. Shallowly inserted amphipathic fusion peptides strongly deform the membrane: (**a**) top view; (**b**) side view (the 3rd peptide is not shown). When fusion peptides are in trans-monolayer configuration, the deformation is minimal: (**c**) top view; (**d**) side view. The polar side surface of the peptide is shown in dark gray; the hydrophobic side surface is shown in red. In panels (**c**), (**d**), the left configurations correspond to predominantly hydrophobic fusion peptides with a small area of the polar surface; the right configurations correspond to fusion peptides, whose hydrophobicity gradually increases from the C- to N-terminal, modeling the fusion peptides of influenza A virus hemagglutinin (HA).

Fusion proteins of type I are trimers. Upon activation, each trimer releases three fusion peptides from hydrophobic pockets. In general, all fusion peptides may (1) miss the target membrane and insert into the viral membrane; (2) reach the target membrane; or (3) be in a state where one or two of them reach the target membrane, while others attack the viral one [24]. In the maximal outcome for each fusion protein, all three fusion peptides incorporate into the cellular membrane. Fusion peptides of influenza and HIV are amphipathic, and generally they should insert into a membrane only shallowly (Figure 1b).

Shallowly inserted peptides are expected to strongly disturb the lipid packing and induce the large deformation of the target membrane (Figure 1b). The deformation energy is minimal when the peptides are able to stand parallel at the distance of about 5 nm [33]. The energy grows steeply as the peptides approach each other; i.e., at short distances, peptide–peptide interactions are strongly repulsive. In the symmetric star-like configuration of the peptides (Figure 1a), the energy is maximal [44]. However, three tightly bound amphipathic peptides may undergo a cooperative transition from surface-bound to trans-monolayer configuration (Figure 1c,d). In such a configuration, deformations should be minimal. The fusion peptides of HIV gp41 are predominantly hydrophobic, and the fusion activity of gp41 is shown to decrease as a result of the increasing polarity of its fusion peptides [11]. Such peptides are expected to completely immerse into the target membrane, forming a barrel-like structure with a majorly hydrophobic outer side surface (Figure 1c,d) left configurations). The free fusion peptide of influenza HA in a membrane attains a boomerang-like shape: its C-terminal part is shallowly inserted into the membrane and is approximately parallel to its surface, while its N-terminal is deeply buried into the lipid monolayer [42]. This allows concluding that the C-terminal part of this fusion peptide is more polar than its N-terminal part. Such peptides are expected to attain a combined configuration: they may hide their N-terminal parts into the lipid monolayer, form a barrel-like structure with a majorly hydrophobic outer side surface, and expose their C-terminal parts to lipid head groups or water (Figure 1c,d right configurations). Note that the configurations illustrated in Figure 1c,d are principally cooperative; separate free fusion peptides cannot spontaneously undergo the transition from surface-bound to trans-monolayer configurations. Three fusion peptides in trans-monolayer configuration can be approximately considered as single cylindrical membrane inclusion.

#### 2.1.2. Anisotropy of Deformation Amplitude

**Figure 2:**
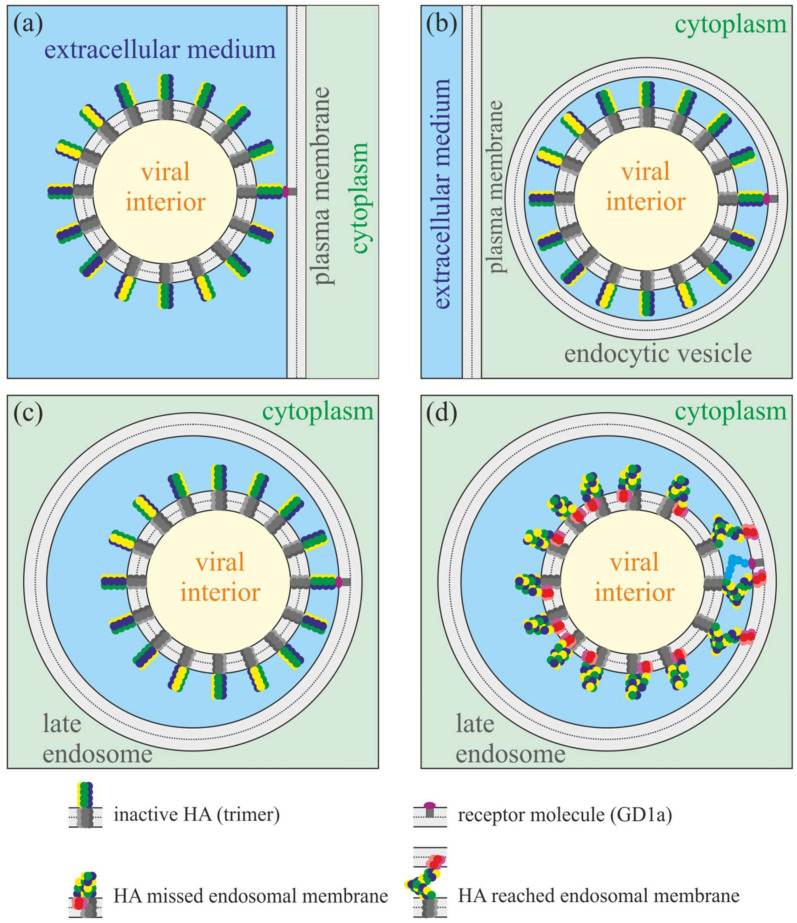
Schematic representation of receptor binding and fusion of influenza virus. Only a few HA molecules can reach the endosomal membrane by their fusion peptides in the course of fusion triggered by low pH in the late endosome. The amplitude of HA-induced deformations of the endosomal membrane should be highly anisotropic. (**a**) Attachment of the viral particle to the receptor molecule (ganglioside GD1a) in the plasma membrane. (**b**) Virion enters the cell in the process of endocytosis. The size of the endocytic vesicle closely fits the size of the viral particle. (**c**) The large endosome fuses with the endocytic vesicle. The virion remains attached to the endosomal membrane due to binding to the receptor molecule. (**d**) Drop of pH in the late endosome triggers the conformation transition of all hemagglutinins. However, only those HAs that are the closest to the receptor molecule can reach the endosomal membrane by their fusion peptides. More distant HAs fold into the post-fusion conformation and insert their fusion peptides into the own viral membrane.

Each viral particle of HIV or influenza has dozens of fusion protein trimers on its surface. However, it seems that only a few of them can reach the target membrane upon activation. The gp41 protein of HIV is triggered by the binding of gp120 to the CD4 receptor and CCR5/CXCR4 co-receptor [45]. Only the gp120 proteins nearest to the cell surface can bind to the receptor, and thus only one to several gp41 fusion proteins of each HIV virion can be activated in the process of HIV-induced membrane fusion [27]. Influenza HA recognizes and binds ganglioside GD1a lipid molecules on a target cell membrane (Figure 2a) [46]. Furthermore, the virion passes into the cell in the process of endocytosis inside the endocytic vesicle (Figure 2b). The size of the vesicle closely fits the size of the viral particle. Later, the endocytic vesicle fuses with a large endosome (Figure 2c) that leads to acidification of the medium around the virion. The drop of pH down to about 5.5 triggers the conformation transition of all HA molecules exposed at the virion’s outer surface. However, as the size of the late endosome substantially exceeds the size of the viral particle, only a few HA molecules, which are the nearest to the bound GD1a, can reach the endosomal membrane by their fusion peptides. For other HAs, the distance between their transmembrane anchor and the endosomal membrane exceeds the full length of the ectodomain. Thus, these HAs have to fold into the post-fusion conformation and insert their fusion peptides into the viral membrane (Figure 2d). Hemagglutinins that reached the endosomal membrane should deform it to a different extent: the closest HAs are the most folded and thus should tilt their fusion peptides more strongly than HAs, which are more distant from the bound GD1a. Thus, the amplitude of deformations induced by HAs in the endosomal membrane should be highly anisotropic: it is the highest around the pole near the receptor molecule and gradually decays as the distance to the pole increases. In a rough approximation, only the strongest deformations in the first layer of fusion peptide trimers around the receptor molecule can be taken into account, while the deformations induced by the next layers (2nd, 3rd, etc.) of fusion peptide trimers can be neglected.

#### 2.1.3. Parameterization of the System

**Figure 3:**
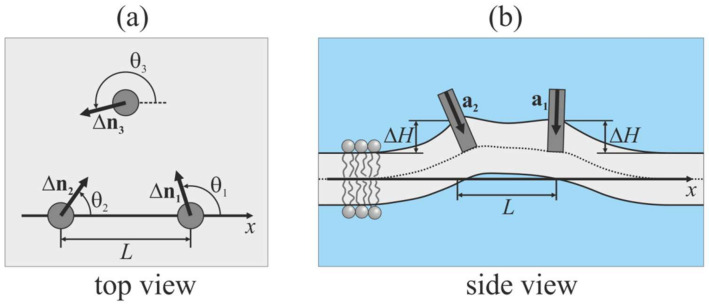
Schematic illustration of configuration of peptides embedded into the upper monolayer of the membrane (shown by light gray color). (**a**) Top view; (**b**) side view. Embedded peptides are shown as gray circles (panel (**a**)) or gray rectangles (panel (**b**)). a is the unit vector directed along the longitudinal peptide axis; Δn is the projection of a onto the *Oxy* plane; *θ* is the angle between the *Ox* axis and Δn; *L* is the distance between peptides; Δ*H* is the vertical position of center of the peptide intersection with the neutral surface of the upper monolayer relative to the position of this surface far from embedded peptides.

To describe the mutual orientations of the peptides, we introduce a Cartesian coordinate system *Oxyz*, in which the *Oxy* plane coincides with the monolayer interface of the planar unperturbed membrane. A monolayer located in the half-space corresponding to positive values of the *z* coordinate is called the upper; the opposite monolayer is called the lower. In case of viral-induced fusion, the upper monolayer corresponds to the contact (proximal or cis-) monolayer of the target membrane; the lower monolayer corresponds to the distal (or trans-) monolayer of the target membrane. The peptides are assumed to be embedded into the upper monolayer; the angle between the unit vector **a**, which is directed along the longitudinal peptide axis (Figure 3b), and the unit normal vector, **N**, to the unperturbed membrane is set at 45°. The projection of vector **a** onto the *Oxy* plane is denoted by Δ**n** (Figure 3a). We direct the *Ox* axis so that it intersects the longitudinal axes of two peptides. For the *i*-th peptide, the angle between the *Ox* axis and Δ**n** is denoted as ***θ****_i_*. We consider configurations of (1) “free” peptides, where only orientation angles ***θ****_i_* are set; and (2) “pulled” peptides, where we additionally fix the value of Δ*H*, which is the vertical position of the center of the peptide intersection with the neutral surface of the upper monolayer relative to the position of this surface far from embedded peptides (i.e., at *x* → ∞).

### 2.2. Two Free Peptide Trimers

**Figure 4:**
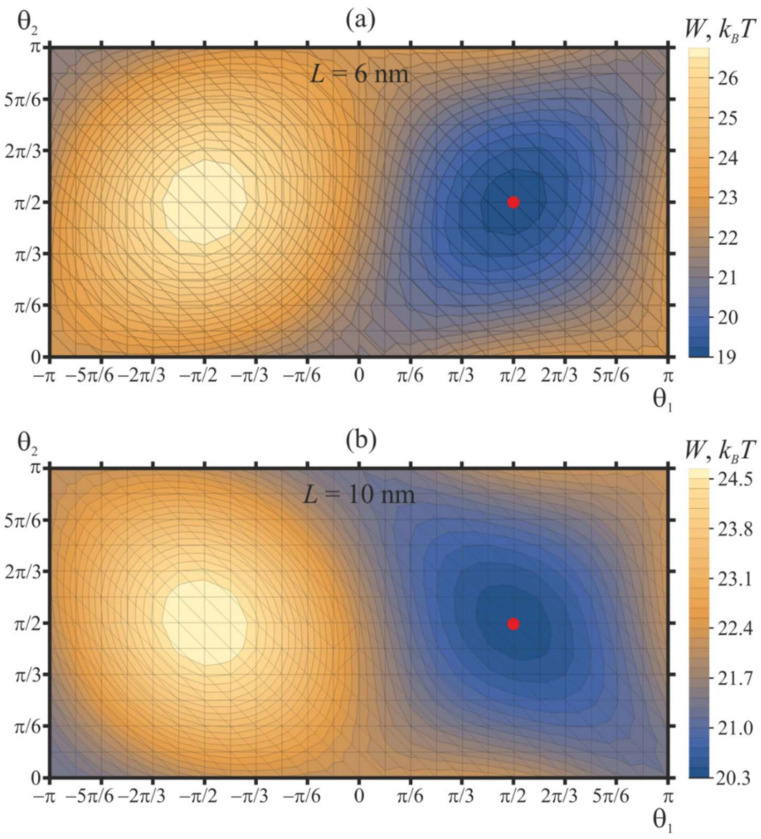
Dependence of membrane elastic energy on the orientation angles of two free peptide trimers separated by (**a**) 6 nm; (**b**) 10 nm. The minimum of the elastic energy (marked by red circle) is at *θ*_1_ = *θ*_2_ = ***π***/2 independent of protein separation.

First, we consider the case of two free peptide trimers, the vertical positions of which are not fixed, and the energy of the system is optimized with respect to Δ*H_i_*. For this case, we set the distance between peptides at either *L* = 6 nm or *L* = 10 nm, we fixed the tilt (out-of-plane) angles of the peptides at 45°, and varied their orientation in-plane angles in the range −*π* ≤ *θ*_1_ ≤ *π*, 0 ≤ *θ*_2_ ≤ *π* with the step of Δ*θ* = *π*/12. At each step, the heights (vertical positions) of peptides were allowed to achieve optimality. The contour plot of the optimal values of the elastic energy of the membrane, *W*, as a function of *θ*_1_, *θ*_2_ is shown in Figure 4.

Independently of the protein separation, the elastic energy has a minimum when the orientation angles of two peptides are equal to *θ*_1_ = *θ*_2_ = *π*/2. We designate such a configuration as “[↑—↑]”, which schematically illustrates the top view of the peptide configuration, and the arrows correspond to in-plane Δ**n** vectors (Figure 3a); these vectors lie in the *Oxy* plane. The tilt (or out-of-plane) angle of the peptide, which is the angle between the **a** and **N** vectors, is always assumed to be fixed and equal to 45°. Due to the symmetry of the system, an identical minimum is presented at *θ*_1_ = *θ*_2_ = −*π*/2 [↓—↓]. The elastic energy is maximal when the orientation angles are equal to *θ*_1_ = −*π*/2, *θ*_2_ = *π*/2 [↓—↑], which is equivalent due to the symmetry, *θ*_1_ = *π*/2, *θ*_2_ = −*π*/2 [↑—↓]. Each minimum (and each maximum) of the elastic energy is surrounded by two pairs of saddle points: (1) the first pair is at *θ*_1_ = *θ*_2_ = 0 [→—→] and *θ*_1_ = *θ*_2_ = *π* [←— ←], (2) the second pair is at *θ*_1_ = 0, *θ*_2_ = *π* [→—←] and *θ*_1_ = *π*, *θ*_2_ = 0 [←—→] (Figure 4). The difference of the membrane elastic energy in the global minimum and in the saddle point with the lowest energy determines the energy barrier of the mutual relative rotation of the peptides—that is, the mutual rotation of the in-plane vectors Δ**n**. For the case of two free peptides separated by *L* = 6 nm (Figure 4a), the lowest-energy saddle points are located at *θ*_1_ = *θ*_2_ = 0 [→—→] and *θ*_1_ = *θ*_2_ = *π* [←— ←]; the height of the energy barrier of peptide rotation is about 2.5 *k_B_T*. This energy barrier corresponds to the transition of the system between the energy minima at *θ*_1_ = *θ*_2_ = *π*/2 [↑—↑] and *θ*_1_ = *θ*_2_ = −*π*/2 [↓—↓] along the optimal trajectory, which passes through the saddle point. The energy barrier of transition between the minima via another pair of saddle points (at *θ*_1_ = 0, *θ*_2_ = *π* [→—←] and *θ*_1_ = *π*, *θ*_2_ = 0 [←—→]) is about 3.7 *k_B_T*. When free peptides are separated by *L* = 10 nm, the lowest-energy saddle points are located at *θ*_1_ = 0, *θ*_2_ = *π* [→—←], and *θ*_1_ = *π*, *θ*_2_ = 0 [←—→] (Figure 4b). The energy barrier of transition between energy minima at *θ*_1_ = *θ*_2_ = *π*/2 [↑—↑] and *θ*_1_ = *θ*_2_ = −*π*/2 [↓—↓] along the optimal trajectory passing through the saddle points *θ*_1_ = 0, *θ*_2_ = *π* [→—←] and *θ*_1_ = *π*, *θ*_2_ = 0 [←—→] is about 1.1. *k_B_T*. The energy barrier along the alternative trajectory passing through saddle points at *θ*_1_ = *θ*_2_ = 0 [→—→] and *θ*_1_ = *θ*_2_ = *π* [←— ←] is about 3 *k_B_T*.

The energy of membrane deformations induced by two free peptides separated by 10 nm is about 20 *k_B_T* in the optimal configuration, i.e., about 10 *k_B_T* per each peptide (Figure 4b). This elastic energy seems to be quite high. However, such magnitude of elastic energy is typical for peptide membrane inclusions. In the work [47], the energy of membrane deformations induced by an amphipathic inclusion is estimated as 2–3 *k_B_T* per 1 nm of the inclusion length. This is in qualitative agreement with the results of the work [33], where it is obtained that the energy of membrane deformations induced by a shallowly inserted 5-nm-long amphipathic peptide is about 7 *k_B_T*. In the work [48], the energy landscape of a single-passing transmembrane peptide in the membrane with coexisting liquid-ordered and liquid-disordered phases is analyzed by means of molecular dynamics. It is shown that the energy penalty of transfer of the peptide from the boundary of the coexisting phases to the bulk liquid-disordered phase can reach about 10 *k_B_T*; the penalty is partially due to the peptide-induced elastic deformations of the membrane. However, experimentally, the peptides are nevertheless registered in the bulk of the liquid-disordered phase, meaning that such an energy penalty cannot prevent the lateral redistribution of the peptides. It is known that small synthetic peptides can effectively induce the fusion of liposomes. In particular, a model system where the fusion of membranes is driven by membrane-anchored synthetic K/E peptides undergoing a coiled-coil transition [49], similar to SNARE-mediated fusion [5], is developed. The authors note that the fusion efficiency will be improved if the membranes are destabilized by shallowly inserted amphipathic peptides inducing local membrane protrusions [50]. The elastic energy of such protrusions is of the order of 10 *k_B_T* [33,47], meaning that the system of even small synthetic peptides is able to develop such mechanical efforts in the course of membrane fusion. The main role of fusogens is to bring merging membranes into close local contact to allow overcoming the hydration repulsion barrier of two lipid bilayers [23]. The characteristic energy of this process is mainly determined by the physicochemical properties of lipid membranes (e.g., polarity, elastic moduli), and thus, it may be expected that the functional mechanisms of most fusogens in various systems should be rather similar; the differences might be dictated by the very specific requirements of fusion parameters in particular system (e.g., the rate or efficiency of fusion).

**Figure 5:**
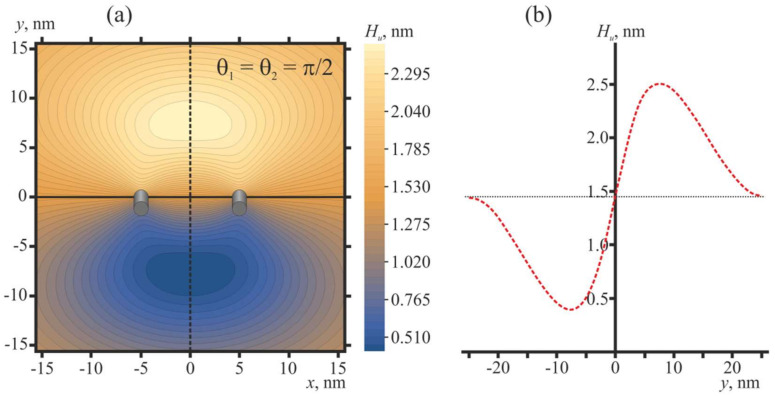
Membrane shape in the vicinity of two free peptide trimers in the optimal configuration (*θ*_1_ = *θ*_2_ = *π*/2). The distance between the peptides is *L* = 10 nm, which corresponds to the conditions of Figure 4b. (**a**) Contour plot of the shape of the neutral surface of the upper monolayer, *H_u_*(*x*, *y*). Peptides are shown as oblique gray cylinders, the centers of which have coordinates *x* = 5 nm, *y* = 0 and *x* = −5 nm, *y* = 0. (**b**) Shape of the neutral surface of the upper monolayer, *H_u_*(0, *y*), along the line *x* = 0 (dashed black line in panel (**a**)) passing in the middle between the peptides. The horizontal dotted line corresponds to the *H_u_* value far from the peptides.

The shape of the membrane in the vicinity of two free peptide trimers in the optimal configuration (*θ*_1_ = *θ*_2_ = *π*/2) is shown in Figure 5.

Oblique free peptides induce an oval-like bulge of the membrane (Figure 5a) of the height of about 1 nm (Figure 5b) with respect to the membrane plane position far from the peptides. For influenza, the distance between two fusing membranes is most probably dictated by HAs folded into a post-fusion state, whose fusion peptides missed the endosomal membrane (Figure 2d); this distance should be about the height of folded ectodomain, i.e., about 10 nm. Thus, the membrane bulge induced by two free peptides in their optimal configuration seems to be too small, and it cannot facilitate membrane fusion to any appreciable extent.

### 2.3. Two Pulled Peptide Trimers

**Figure 6:**
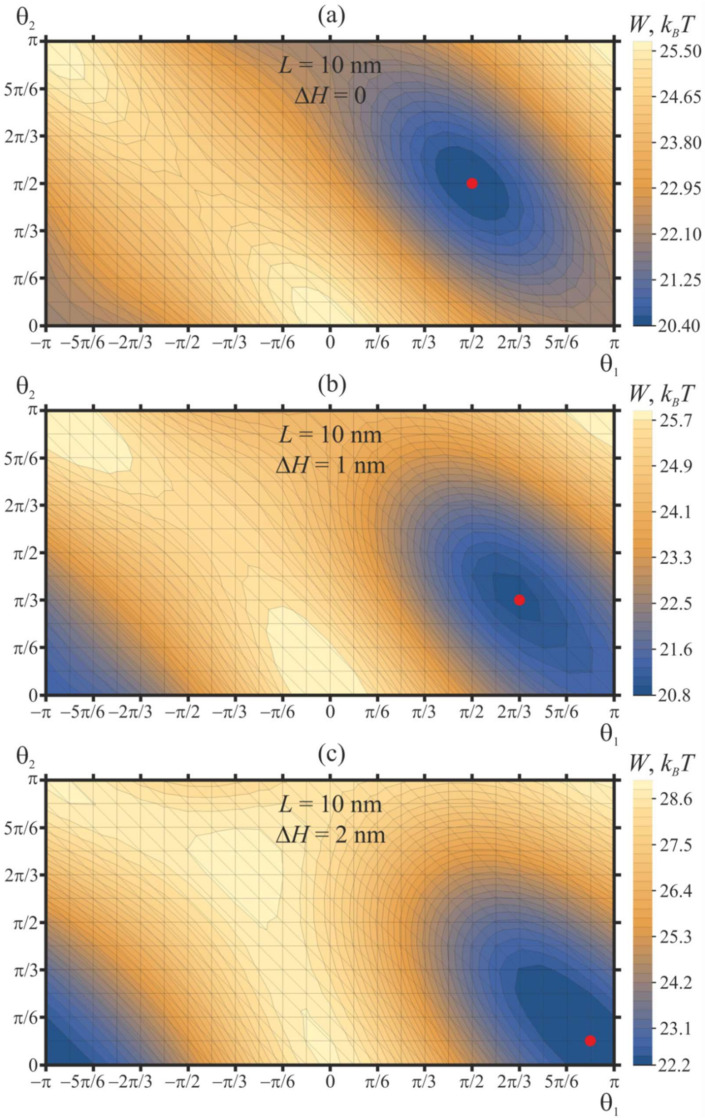
Dependence of membrane elastic energy on the orientation angles of two pulled peptide trimers, whose vertical positions are fixed at (**a**) Δ*H* = 0; (**b**) Δ*H* = 1 nm; (**c**) Δ*H* = 2 nm. The minima of the elastic energy (marked by the red circle) are at (**a**) *θ*_1_ = *θ*_2_ = *π*/2 for Δ*H* = 0; (**b**) *θ*_1_ = 2*π*/3, *θ*_2_ = *π*/3 for Δ*H* = 1 nm; (**c**) *θ*_1_ = 11*π*/12, *θ*_2_ = *π*/12 for Δ*H* = 2 nm. The minima shifts toward the symmetric configuration of *θ*_1_ = *π*, *θ*_2_ = 0 as Δ*H* increases.

We consider the case of two pulled peptide trimers, whose vertical positions are fixed at certain values of Δ*H*. For this case, the distance between the peptides is set at *L* = 10 nm, and their orientation angles are varied in the range −*π* ≤ *θ*_1_ ≤ *π*, 0 ≤ *θ*_2_ ≤ *π* with the step of Δ*θ* = *π*/12. Contour plots of the elastic energy of the membrane, *W*, as a function of *θ*_1_, *θ*_2_ are shown in Figure 6. In case of pulled peptides, a structure of the energy surface W(*θ*_1_, *θ*_2_) is qualitatively similar to that of free peptides: there are periodic series of elastic energy minima and maxima, each of which is surrounded by two pairs of saddle points. When the vertical positions of the peptides are fixed in the plane of an unperturbed upper monolayer (Δ*H* = 0), the global minimum of the elastic energy is located at *θ*_1_ = *θ*_2_ = *π*/2 [↑—↑] and *θ*_1_ = *θ*_2_ = −*π*/2 [↓—↓] (Figure 6a), exactly as in the case of free peptides (Figure 4b). However, the positions of the energy maxima (*θ*_1_ = *θ*_2_ = 0 [→—→] and *θ*_1_ = *θ*_2_ = *π* [←—←]) as well as of the saddle points (*θ*_1_ = −*π*/2, *θ*_2_ = *π*/2 [↓—↑] and *θ*_1_ = 0, *θ*_2_ = *π* [→—←]) shift substantially. The energy barrier of the transition between the energy minima via the lowest-energy saddle point (which is at *θ*_1_ = 0, *θ*_2_ = *π* [→—←]) is about 1.7 *k_B_T* for the case of Δ*H* = 0 (Figure 6a). The energy barrier along the trajectory passing through the alternative saddle point at (*θ*_1_ = −*π*/2, *θ*_2_ = *π*/2 [↓—↑] is substantially higher: about 4.4. *k_B_T*.

As the peptides are pulled vertically, the energy landscape *W*(*θ*_1_, *θ*_2_) becomes more rough: the depth of the energy minima increases, and the height of the energy barriers for the mutual relative rotation of peptides grows (Figure 6b). For the case of Δ*H* = 1 nm, the minimum of the elastic energy is located at *θ*_1_ = 2*π*/3, *θ*_2_ = *π*/3, while the maximum is located at *θ*_1_ = *θ*_2_ = 0. The difference of the elastic energy in the global minimum and the lowest-energy saddle point at *θ*_1_ = 0, *θ*_2_ = *π* is about 3.5 *k_B_T*, meaning that the mutual relative rotation of the peptides is substantially hampered when the peptides are pulled out of the plane of an unperturbed membrane.

When the peptides are pulled further, up to the value of Δ*H* = 2 nm (Figure 6c), the global minimum of the elastic energy appears at *θ*_1_ = 11*π*/12, *θ*_2_ = *π*/12. This allows tracing the tendency of the tilted peptides to orient into the highly symmetric configuration of *θ*_1_ = *π*, *θ*_2_ = 0 [←—→] as they are progressively pulled out of the plane of an unperturbed membrane—compare the positions of the global energy minimum for Δ*H* = 0 (Figure 6a), Δ*H* = 1 nm (Figure 6b), and Δ*H* = 2 nm (Figure 6c). Simultaneously, the energy values in the global maxima and saddle points of the elastic energy converge: for the case of Δ*H* = 2 nm, they differ by about 0.3 *k_B_T* only (Figure 6c). The height of the energy barrier of transition between the energy minima via the lowest-energy saddle point grows sharply: for Δ*H* = 2 nm, this energy barrier is about 6.5 *k_B_T*. This means that upon the pulling of the tilted peptides, the energy stability of the attained symmetric configuration *θ*_1_ = *π*, *θ*_2_ = 0 [←—→] increases.

**Figure 7:**
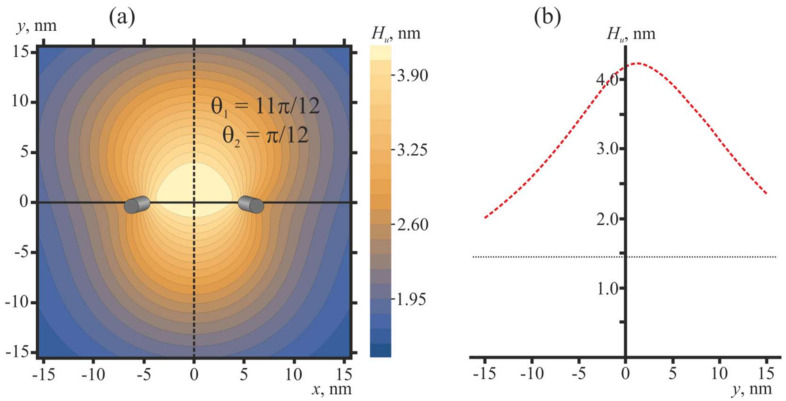
Membrane shape in the vicinity of two pulled peptide trimers (Δ*H* = 2 nm) in the optimal configuration (***θ***_1_ = 11***π***/12, ***θ***_2_ = ***π***/12) that corresponds to the conditions of Figure 6c. (**a**) Contour plot of the shape of the neutral surface of the upper monolayer, *H_u_*(*x*, *y*). Peptides are shown as oblique gray cylinders, the centers of which have coordinates *x* = 5 nm, *y* = 0, and *x* = −5 nm, *y* = 0. (**b**) Shape of the neutral surface of the upper monolayer, *H_u_*(0, *y*), along the line *x* = 0 (dashed black line in panel (**a**)) passing in the middle between the peptides. The horizontal dotted line corresponds to the *H_u_* value far from the peptides.

The shape of the membrane in the vicinity of two pulled peptide trimers (Δ*H* = 2 nm) in the optimal configuration (***θ***_1_ = 11***π***/12, ***θ***_2_ = ***π***/12) is shown in Figure 7.

Oblique pulled peptide trimers induce a bulge of the membrane (Figure 7a) with a height of about 2.8 nm (Figure 7b) with respect to the membrane plane position far from the peptides.

### 2.4. Three Peptide Trimers

**Figure 8:**
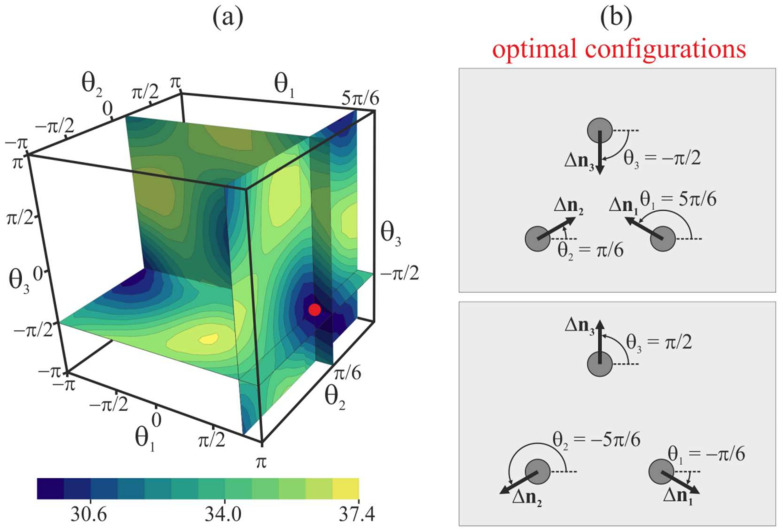
(**a**) Dependence of membrane elastic energy on the orientation angles of three free peptide trimers separated by 10 nm. The minimum of the elastic energy (marked by red circle) is at *θ*_1_ = 5*π*/6, *θ*_2_ = *π*/6, *θ*_3_ = −*π*/2, and at *θ*_1_ = −*π*/6, *θ*_2_ = −5*π*/6, *θ*_3_ = *π*/2. (**b**) Top view of optimal configurations of the peptides.

Furthermore, we consider the case of three free peptide trimers, the vertical positions of which are not fixed; i.e., the energy of the system is optimized with respect to Δ*H*. For this case, the distance between the peptides is set at *L* = 10 nm, and their orientation angles *θ*_1_, *θ*_2_, and *θ*_3_ are varied in the range from −*π* to *π* with the step of Δ*θ* = *π*/6. The plot of the elastic energy of the membrane, *W*, as a function of *θ*_1_, *θ*_2_, and *θ*_3_ is shown in Figure 8a. Peptides are situated in the vertices of a right triangle, the side of which has the length *L* = 10 nm. The global minima of the elastic energy are located in *θ*_1_ = 5*π*/6, *θ*_2_ = *π*/6, *θ*_3_ = −*π*/2, and *θ*_1_ = −*π*/6, *θ*_2_ = −5*π*/6, *θ*_3_ = *π*/2 (Figure 8b); the minimal energy values are the same for these two minima and equal to 29.7 *k_B_T*. The minima are connected by a low-energy valley; the energy barrier of passing from one minimum to another along the valley is about 0.3 *k_B_T*. In case of three peptides, the highly symmetric configurations shown in Figure 8b are attained spontaneously, without the need for pulling the peptides out of the membrane plane, although the energy barrier separating the minima is relatively low. Some symmetry is introduced into the system by placing the peptides at the vertices of the right triangle. Thus, the peptide positional symmetry may induce the orientational symmetry. The optimal vertical position of three free peptide trimers with respect to the *z*-coordinate of the neutral surface of the upper monolayer far from the peptides is Δ*H* ≈ 2.5 nm for the optimal configuration *θ*_1_ = 5*π*/6, *θ*_2_ = *π*/6, *θ*_3_ = −*π*/2 (i.e., the membrane bulges upwards) and Δ*H* ≈ −2.5 nm for the optimal configuration *θ*_1_ = −*π*/6, *θ*_2_ = −5*π*/6, *θ*_3_ = *π*/2, i.e., the membrane bulge is directed downwards.

Furthermore, we obtained the elastic energy of the membrane, *W*, as a function of *θ*_1_, *θ*_2_, *θ*_3_ for the case of fixed vertical positions Δ*H* of three peptide trimers. When Δ*H* = 0, the energy landscape is virtually the same as in the case of free peptides: there are two global minima of the energy of the same value of 33 *k_B_T*. The optimal configurations of the peptides coincide with those of the free peptides, as schematically illustrated in Figure 8b. The energy barrier between the global energy minima along the low-energy valley is about 1.3 *k_B_T*. When the peptides are pulled upward by Δ*H* = 1 nm, the energy minimum at *θ*_1_ = −*π*/6, *θ*_2_ = −5*π*/6, *θ*_3_ = *π*/2 disappears as the energy of this configuration grows up by about 7.5 *k_B_T*. The minimal energy value in the only remaining optimal configuration *θ*_1_ = 5*π*/6, *θ*_2_ = *π*/6, *θ*_3_ = −*π*/2 is 30.3 *k_B_T*. The minimal energy barrier of the peptides rotation by a complete period *θ*_1_ → 5*π*/6 + 2*π*, *θ*_2_ → *π*/6 + 2*π*, *θ*_3_ → −*π*/2 + 2*π* is about 5.5 *k_B_T*, meaning that in case of pulled peptides by Δ*H* = 1 nm, the optimal configuration of the peptides is rather stable with respect to thermally accessible rotations. When the peptides are pulled upward by Δ*H* = 2 nm, the only energy minimum at *θ*_1_ = 5*π*/6, *θ*_2_ = *π*/6, *θ*_3_ = −*π*/2 becomes deeper: the corresponding value is 29.8 *k_B_T*. The minimal energy barrier of the peptides’ rotation by a complete period *θ*_1_ → 5*π*/6 + 2*π*, *θ*_2_ → *π*/6 + 2*π*, *θ*_3_ → −*π*/2 + 2*π* is about 8.8 *k_B_T*, meaning that in case of pulled peptides by Δ*H* = 2 nm, the thermal rotation of the peptides is practically prohibited.

**Figure 9:**
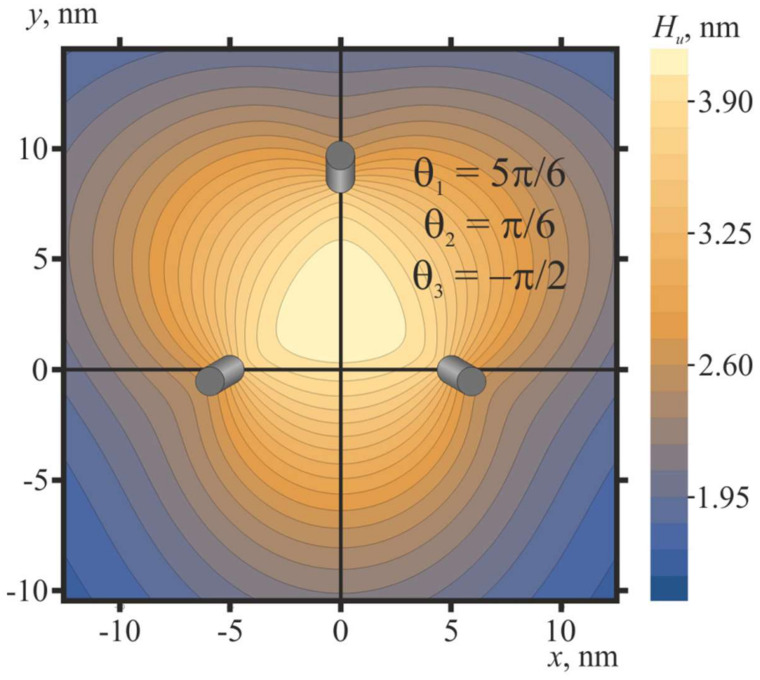
Membrane shape in the vicinity of three pulled peptide trimers (Δ*H* = 2 nm) in the optimal configuration (*θ*_1_ = 5*π*/6, *θ*_2_ = *π*/6, *θ*_3_ = −*π*/2): the contour plot of the shape of the neutral surface of the upper monolayer, *H_u_*(*x*, *y*). Trimers are shown as oblique gray cylinders, the centers of which have coordinates *x* = 5 nm, *y* = 0; *x* = −5 nm, *y* = 0; *x* = 0, *y* = 75^1/2^ ≈ 8.7.

The shape of the membrane in the vicinity of three pulled peptide trimers (Δ*H* = 2 nm) in the optimal configuration (*θ*_1_ = 5*π*/6, *θ*_2_ = *π*/6, *θ*_3_ = −*π*/2) is shown in Figure 9. Oblique pulled peptide trimers induce a bulge of the membrane (Figure 9) of the height of about 2.8 nm with respect to the membrane plane position far from the peptides. Note that the calculated membrane shape for Δ*H* = 2 nm is virtually the same as in the case of three free peptide trimers in the optimal configuration *θ*_1_ = 5*π*/6, *θ*_2_ = *π*/6, *θ*_3_ = −*π*/2.

## 3. Discussion

The fusion of biological membranes requires the close contact of two lipid bilayers to overcome the hydration repulsion between them, which is minimized by decreasing their area of contact at a fusion site. Further remodeling is accompanied with strong deformations of the lipid matrix [9,23]. Thus, membrane merger is an energetically expensive process, prohibiting spontaneous fusion. In biological systems, fusion is allowed because of the activity of specific fusion proteins [4,5]. The fusion of membranes of enveloped viruses with cellular membranes is a key stage of the viral lifecycle. Viral fusion proteins are classified into three classes. The fusion proteins of influenza and HIV (hemagglutinin and gp120/gp41, respectively) belong to class I, and both of them form trimers [7]. Several studies suggest that these proteins should act as a team to accomplish the viral fusion [24,26,27]. Nevertheless, the nature and necessity of such orchestrated functioning of fusion proteins still remain obscure.

In the present work, we considered the possible mechanism of the cooperativity between viral fusion proteins. We demonstrated that the elastic membrane deformations induced by fusion peptides incorporated into the cellular membrane could drive the orientation of the peptides into a fusion rosette possessing a central symmetry. The formation of the symmetric structure requires either a symmetric spatial positioning of the peptides (e.g., at vertices of a right triangle; see Figure 8) or pulling the peptides out of the membrane plane (Figure 6). On the contrary, free peptides arrange into the structure of a lower symmetry, which seems to be incompetent of membrane fusion (Figure 4). Earlier, the membrane-mediated interaction of obliquely incorporated membrane inclusions modeling fusion proteins was analyzed in the work [31]. This work considers effectively transmembrane inclusions, as the peptide-free monolayer (the lower one in our model) was not explicitly introduced. Besides, the work considers the case of two free peptides, and it is obtained that their equilibrium orientation corresponds to *θ*_1_ = *θ*_2_ = *π*/2 [↑—↑] in our notation. Our results perfectly agree with this finding of the work [31] (Figure 4).

In case of the hemagglutinin-mediated fusion of membranes, the fusion peptides are most probably both symmetrically positioned due to the high surface density of the fusion proteins, and they are subject to a pulling force generated in the course of the conformational rearrangement of hemagglutinin [8,12]. These two factors could ensure the formation of a highly cooperative ring-like fusion rosette.

In case of HIV, the fusion may be catalyzed by a few Env proteins, down to a single protein trimer [27]. The trimeric structure of gp120/gp41 suggests that three fusion peptides may simultaneously incorporate into the target membrane. However, if the peptides are incorporated symmetrically (e.g., they are located at vertices of a right triangle), in the target membrane, they will drive a formation of the bulge just opposing the gp120/gp41 trimer. This may hinder the membrane fusion due to steric restrictions. Along with fusion, the Env proteins are responsible for the reception of the virus, i.e., its binding to the cellular plasma membrane [51]. The gp120 protein recognizes the CD4 receptor and CXCR4 or CCR5 co-receptor [52,53], which are transmembrane proteins [54]. Receptor binding triggers the conformational rearrangement of the Env glycoprotein. This type of triggering additionally limits the number of simultaneously activated gp120/gp41 trimers, which is due to relatively low surface density of CD4 molecules in the plasma membrane of T-lymphocytes. This is in contrast to the case of influenza virus, in which hemagglutinin activation is triggered by the decrease of pH in the late endosome, i.e., all hemagglutinin trimers of the virion are activated almost simultaneously. Upon binding to CD4 and CXCR4 or CCR5, the gp120 subunit of the Env protein shifts laterally, which should lead to a break of the central symmetry. We hypothesize that in case of HIV, the fusion rosette in the target membrane might consist of three fusion peptides of a single gp41 trimer, standing in the trans-monolayer configuration, and of the shifted and tilted CD4 receptor and/or co-receptors bound to gp120. This might lead to the formation of the centrally symmetric structure, whose axis of symmetry is laterally shifted with respect to the axis of the gp41 trimer. Thus, no steric restriction would arise in the course of fusion: in such a configuration, the bulge in the target membrane would be formed between the trimer axis and the CD4 receptor, rather than exactly on the axis of the gp41 trimer.

## 4. Materials and Methods

We consider elastic deformations of a lipid membrane basing on the continuum theory of elasticity developed in the work [55]. Deformations are assumed to be small, and their energy is calculated in quadratic approximation. The ground state of the membrane is assumed to be flat. We introduce a Cartesian coordinate system *Oxyz* with the *Oxy* plane coinciding with the intermonolayer surface of the flat unperturbed membrane. A monolayer located in the half-space corresponding to the positive values of the *z* coordinate is called the upper; the opposite monolayer is called the lower. Values related to the upper monolayer are denoted by index “*u*”; values related to the lower monolayer are denoted by index “*l*”. The average orientation of lipid molecules is described by a field of unit vectors **n** called directors. The field of directors is assumed to be set on some surface referred to as a dividing surface, which lies inside the monolayer parallel to its external (polar) surface. The shape of the dividing surface is characterized by a function *H*(*x*, *y*), which is equal to the distance from the plane *Oxy* to the dividing surface measured along the direction of the *Oz* axis. The shape of the intermonolayer surface is characterized by a function *M*(*x*, *y*), which is equal to the distance from the plane *Oxy* to the intermonolayer surface measured along the direction of the *Oz* axis. We consider the following deformations of a lipid monolayer [33,56,57]: (1) splay, which is characterized by a divergence of the director along the dividing surface, div(**n**); (2) tilt, which is characterized by a tilt vector **t** = **n**/(**nN**) − **N** ≈ **n** − **N**, where **N** is the unit normal vector to the dividing surface; (3) lateral stretching–compression, which is characterized by a relative change in the area per molecule at the dividing surface, *α* = (*a* − *a*_0_)/*a*_0_ (*a*, *a*_0_ are the current and initial area per molecule at the dividing surface, respectively); (4) lateral tension, which is characterized by a deviation of the dividing surface from a horizontal plane, **grad**(*H*); (5) saddle splay, which is characterized by $K=\frac{\partial n_{x}}{\partial x}\frac{\partial n_{y}}{\partial y}-\frac{\partial n_{x}}{\partial y}\frac{\partial n_{y}}{\partial x}$, where *n_x_*, *n_y_* are projections of the director onto *Ox*, *Oy* axes, respectively; and (6) twist, which is characterized by **rot**(**n**). All deformations are deemed small, and the quadratic approximation of the elastic energy is considered; the contributions of higher orders (and the influence of thermal fluctuations of the membrane arising from them) are neglected. It is shown in [58] that the quadratic approximation describes well the membrane deformations up to fairly strong deformations, arising on the spatial scale comparable with the size of individual lipid molecules. The elastic energy of a lipid monolayer in such approximations can be written as [33,36]:

(1) $$\begin{array}{c} W=\int dS\left(\frac{B}{2}{\left(\mathrm{div}n+J_{0}\right)}^{2}-\frac{B}{2}J_{0}^{2}+\frac{K_{t}}{2}t^{2}+\frac{\sigma }{2}{\left(grad\;H\right)}^{2}\right.+\\ \left.+\frac{K_{a}}{2}\alpha^{2}+K_{G}K+\frac{K_{\mathrm{rot}}}{2}{\left(rot\;n\right)}^{2}\right), \end{array}$$

where *B*, *K_t_*, *K_a_*, and *K_G_* are the splay, tilt, lateral stretching–compression, and Gaussian (saddle splay) moduli of the monolayer, respectively; *K*_rot_ is the twist modulus (*K*_rot_ = 0 for axially symmetric configurations or cases of **t** = **0** corresponding to a smectic liquid crystal without internal structure); *σ* is the lateral tension (per monolayer); and *J*_0_ is the spontaneous curvature of the monolayer; the integration is performed over the dividing surface of the monolayer. We relate deformations and elastic moduli to the specific dividing surface called the neutral surface, where deformations of splay and lateral stretching–compression are energetically decoupled, formally leading to the absence of the corresponding cross-term in the energy functional Equation (1). Such a surface is experimentally shown to exist and lie in the region of the junction of the polar heads and hydrophobic tails of lipids, which is about 0.7 nm deep from the outer polar surface of the monolayer [59]. We also assume that the lipid monolayer is locally volumetrically incompressible, meaning that each element preserves its original volume upon deformation. This assumption is justified by the large bulk modulus of the lipid membrane, which is approximately 10^4^ *k_B_T*/nm^3^ according to [60]. Local incompressibility imposes restrictions on possible deformations, which can be written with the required accuracy as follows [55]:

(2) $$H_{u}-M=h-\frac{h^{2}}{2}\mathrm{div}\left(n_{u}\right)-h\alpha_{u},\;M-H_{l}=h-\frac{h^{2}}{2}\mathrm{div}\left(n_{l}\right)-h\alpha_{l},$$

where *h* is the thickness of the hydrophobic part of the undeformed lipid monolayer. Equation (2) implies that the distance between the neutral surface of the monolayer and the intermonolayer surface, |*H* − *M*|, is equal to the hydrophobic length of a lipid, whose deviation from its equilibrium value *h* is determined by the deformations experienced by the lipid. For small deformations $n={\left(n_{x}\left(x,y\right),n_{y}\left(x,y\right),\mp 1\right)}^{T}$, $N={\left(\pm \partial H\left(x,y\right)/\partial x,\pm \partial H\left(x,y\right)/\partial y,\mp 1\right)}^{T}$, where upper signs correspond to the upper monolayer and lower signs correspond to the lower monolayer, *T* means transposition, while subscripts “*x*” and “*y*” designate the projection of the vector to the corresponding axis. Taking these relations into account, combining Equations (1) and (2), with the required accuracy, we can write the energy functional of the bilayer membrane as follows:

(3) $$\begin{array}{c} W=\int dS_{u}\left(\frac{B}{2}{\left(\mathrm{div}n_{u}+J_{0}\right)}^{2}-\frac{B}{2}J_{0}^{2}+\frac{K_{t}}{2}{\left(n_{u}-grad\;H_{u}\right)}^{2}+\frac{\sigma }{2}{\left(grad\;H_{u}\right)}^{2}\right.+\\ \left.+\frac{K_{a}}{2h^{2}}{\left(h-\frac{h^{2}}{2}\mathrm{div}n_{u}+M-H_{u}\right)}^{2}+K_{G}K_{u}+\frac{K_{\mathrm{rot}}}{2}{\left(rot\;n_{u}\right)}^{2}\right)+\\ +\int dS_{l}\left(\frac{B}{2}{\left(\mathrm{div}n_{l}+J_{0}\right)}^{2}-\frac{B}{2}J_{0}^{2}+\frac{K_{t}}{2}{\left(n_{l}+grad\;H_{l}\right)}^{2}+\frac{\sigma }{2}{\left(grad\;H_{l}\right)}^{2}\right.+\\ \left.+\frac{K_{a}}{2h^{2}}{\left(h-\frac{h^{2}}{2}\mathrm{div}n_{l}-M+H_{l}\right)}^{2}+K_{G}K_{l}+\frac{K_{\mathrm{rot}}}{2}{\left(rot\;n_{l}\right)}^{2}\right). \end{array}$$

To obtain quantitative results, we used elastic parameters that are typical for membranes made from dioleoylposphatidylcholine (DOPC): splay modulus *B* = 0.42 × 10^−19^ J ≈ 10 *k_B_T* [61]; tilt modulus *K_t_* = 40 mN/m ≈ 10 *k_B_T*/nm^2^ [55]; lateral compression–stretching modulus *K_a_* = 133 mN/m ≈ 32 *k_B_T*/nm^2^ [61]; Gaussian modulus *K_G_* = −0.5*B* = −5 *k_B_T* [62]; lateral tension *σ* = 0.1 mN/m ≈ 0.025 *k_B_T*/nm^2^; spontaneous curvature *J*_0_ = −0.091 nm^−1^ [63]; thickness of the hydrophobic part of the monolayer *h* = 1.45 nm [61,64,65]; twist modulus *K*_rot_ = *B*/2 = 5 *k_B_T* [33]. All values are given per one monolayer.

The energy functional of Equation (3) should be supplemented by the general boundary conditions of the unperturbed membrane at infinity (far from the inclusions):

(4) $$n_{u,l}\left(\infty \right)={\left(0,0,\mp 1\right)}^{T},\;M\left(\infty \right)=0,\;H_{u}\left(\infty \right)=h,\;H_{l}\left(\infty \right)=-h.$$

The directors and neutral surfaces of the upper and lower monolayers are supposed to be continuous everywhere except for the inclusions. Besides, specific boundary conditions should be set at each membrane inclusion (the part of the fusion peptide incorporated into the membrane). For cylindrical inclusion of a radius *r*_0_ embedded vertically into the upper monolayer, we use the following conditions:

(5) $$\begin{array}{l} n_{ux}(\Gamma )=0,\\ n_{uy}(\Gamma )=0,\\ H_{u}\left(\Gamma \right)=H_{0}, \end{array}$$

where Γ is the contour of the fusion peptide boundary at the neutral surface of the upper monolayer (i.e., Γ is approximately a circle of the radius *r*_0_); *n_x_* and *n_y_* are the corresponding components of the director vector; and *H*_0_ is the constant obtained from the minimization of the total energy. The actual value of the boundary director is determined by the various interactions of the peptide with the lipid environment; therefore, the boundary conditions (5) should be considered as qualitative estimates rather than strict quantitative relations. It is important to note that the vertically incorporated inclusion does not deform the membrane, as in this case, the director orientation coincides with that of the unperturbed membrane, and minimization of the total energy with respect to *H*_0_ yields *H*_0_ = *h*, which also corresponds to the state of the unperturbed membrane. In case of inclusion tilt with respect to the normal to the neutral surface of the upper monolayer, the boundary directors deviate from the direction of the *Oz* axis by the vector Δ**n** lying in the *Oxy* plane:

(6) $$n_{ux}\left(\Gamma \right)=\Delta n_{x},n_{uy}\left(\Gamma \right)=\Delta n_{y}.$$

The absolute value of the vector of the peptide tilt, |Δ**n**|, is considered constant, which is determined by the complex interplay of the pulling force applied by the fusion protein, hydrophobic and electrostatic interactions, etc. The components of the inclusion tilt vector can be written as:

(7) $$\Delta n_{x}=\left|\Delta n\right|\cos\theta ,\;\Delta n_{y}=\left|\Delta n\right|\sin\theta ,$$

where *θ* is the angle between Δ**n** and the *Ox* axis. Each point on the boundary contour of the inclusion at the neutral surface can be set by its radius vector **r**. Let us denote the radius vector of the center of the boundary contour as **r_0_**. In such notations, the third boundary condition in (5) changes as follows for the case of tilted inclusion:

(8) $$H_{u}(\Gamma )=H_{0}+\Delta r\Delta n,$$

where Δ**r** = **r** − **r_0_**.

To calculate the membrane deformation energy in the presence of several fusion peptide trimers inserted into the membrane, we use the relations (6)–(8) as boundary conditions at the boundary of each inclusion, considering the same values of |Δ**n**| and different angles *θ* for different peptides. As the energy functional (3) is quadratic on deformations, the total elastic energy is proportional to |Δ**n**|^2^. For definiteness, we take the value |Δ**n**| = 0.7, which corresponds to the peptide tilt angle of about 45°, and set *r*_0_ = 0.65 nm. Generally, the considered system of two or three obliquely oriented inclusions lacks rotational or translational symmetry. In this case, the variation of the energy functional results in Euler–Lagrange partial differential equations, which cannot be solved analytically under the specified boundary conditions in Equations (4) and (6)–(8). Thus, we numerically minimized the energy functional (3) with the boundary conditions in Equations (4) and (6)–(8) using the finite element method with an adaptive mesh. In order to eliminate the effect of the finite size of the mesh, we calculated at least five values of the system energy for sequentially decreasing meshes, and then applied quadratic extrapolation to zero size of the computational mesh, similarly to the works [33,36].

## 5. Conclusions

Two possible mechanisms of fusion protein action are traditionally proposed. One of them is based on the assumption that the incorporated fusion peptides modify the elastic properties of the target membrane—first of all, its spontaneous curvature [66,67]. It is thought that the altered spontaneous curvature in the ring-like zone of the fusion rosette might be responsible for the formation of the bulge on the target membrane; the fusing membranes come into close contact at the top of the bulge, which substantially facilitates the merger of membranes. According to the second mechanism, instead of modifying the target membrane, the fusion proteins induce bending torques, yielding the formation of bulges and generating pulling forces, directly and mechanically bringing two merging membranes into close contact [3]. Based on the results of the present work, we can argue that the second mechanism provides the formation of highly symmetric fusion rosette due to both tilting the fusion peptides and pulling them out of the plane of the target membrane, thus ensuring cooperation of the mechanical efforts of fusion proteins. On the contrary, the first mechanism uses the symmetry of the fusion rosette to explain the formation of the bulges on fusing membranes, rather than providing an explanation of the symmetry origin. Thus, we conclude that the direct mechanical activity of fusion proteins should drive the merger of membranes more reliably as compared to the local modification of the elastic properties of the target membrane by the incorporated fusion peptides. This activity could result from the concerted action of different subunits of fusion protein, placing the bound cell receptor molecule as an active player in the scene. The latter assumption could be the answer to the differences in the cooperativity of fusion proteins of influenza and HIV: we need 3–9 pH-activated hemagglutinin trimers for effective fusion, while a single receptor-bound gp120/gp41 trimer of HIV would be enough.
